# GPS-PAIL: prediction of lysine acetyltransferase-specific modification sites from protein sequences

**DOI:** 10.1038/srep39787

**Published:** 2016-12-22

**Authors:** Wankun Deng, Chenwei Wang, Ying Zhang, Yang Xu, Shuang Zhang, Zexian Liu, Yu Xue

**Affiliations:** 1 Key Laboratory of Molecular Biophysics of Ministry of Education, College of Life Science and Technology and the Collaborative Innovation Center for Brain Science, Huazhong University of Science and Technology, Wuhan, Hubei 430074, China; 2 Sun Yat-sen University Cancer Center, Guangzhou, Guangdong 510060, China

## Abstract

Protein acetylation catalyzed by specific histone acetyltransferases (HATs) is an essential post-translational modification (PTM) and involved in the regulation a broad spectrum of biological processes in eukaryotes. Although several ten thousands of acetylation sites have been experimentally identified, the upstream HATs for most of the sites are unclear. Thus, the identification of HAT-specific acetylation sites is fundamental for understanding the regulatory mechanisms of protein acetylation. In this work, we first collected 702 known HAT-specific acetylation sites of 205 proteins from the literature and public data resources, and a motif-based analysis demonstrated that different types of HATs exhibit similar but considerably distinct sequence preferences for substrate recognition. Using 544 human HAT-specific sites for training, we constructed a highly useful tool of GPS-PAIL for the prediction of HAT-specific sites for up to seven HATs, including CREBBP, EP300, HAT1, KAT2A, KAT2B, KAT5 and KAT8. The prediction accuracy of GPS-PAIL was critically evaluated, with a satisfying performance. Using GPS-PAIL, we also performed a large-scale prediction of potential HATs for known acetylation sites identified from high-throughput experiments in nine eukaryotes. Both online service and local packages were implemented, and GPS-PAIL is freely available at: http://pail.biocuckoo.org.

As one of the most important and ubiquitous post-translational modifications (PTMs) in proteins, the lysine acetylation catalyzed by histone acetyltransferases (HATs) or lysine acetyltransferases (KATs) reversibly regulates a large number of biological processes, such as transcriptional regulation, metabolism and autophagy^1^,^2^,^3^,^4^,^5^,^6^,^7^. The dysregulation of site-specific HAT-substrate relations is frequently associated with human diseases such as cancers^2^,^3^,^8^,^9^. In eukaryotes, numerous HATs have been classified into three major families including p300/CBP, GCN5-related N-acetyltransferases (GNATs) and MYST proteins^1^,^2^,^3^,^10^,^11^. Different HATs can recognize overlapping but distinct substrates^1^,^11^,^12^. Most HATs exist in multisubunit complexes *in vivo* by physically interacting with non-catalytic proteins, which are also involved in recognizing substrates and synergistically determine the specificity together with HATs^2^,^3^. In this regard, the identification of HAT-specific acetylation sites in proteins is fundamental for understanding the molecular mechanisms and regulatory roles of lysine acetylation.

Previously, systematic identification of protein acetylation sites or “acetylome” was a great challenge, due to the technical limitation^4^,^13^. For example, in 2006, Kim *et al*. used an anti-acetyllysine antibody to purify acetyl-peptides and only detected 388 acetylation sites of 195 proteins from human HeLa cells and mouse liver mitochondria^4^. Recently, advances in the development of high-throughput mass spectrometry (HTP-MS) and highly potent anti-acetyllysine antibodies have greatly improved the acetylomic profiling. For example, in 2009, Choudhary *et al*. identified ~3,600 lysine acetylation sites in 1,750 proteins from a human acute myeloid leukemia cell line^7^. Later, Zhao *et al*. detected >1,300 acetyl-peptides of 1,047 proteins human liver tissues, and further demonstrated a number of metabolic enzymes to be regulated by acetylation^5^. More recently, Svinkina *et al*. totally identified and quantified more than 10,000 acetyl-peptides in over 3,000 proteins from Jurkat cells treated with or without suberoylanilide hydroxamic acid (SAHA)^14^. In our database of compendium of protein lysine modifications (CPLM), we manually curated known acetylation information and totally collected 20,088 acetylated substrates with 58,563 sites^15^. Although more and more acetylation sites were experimentally characterized, the regulatory HATs for most of sites remain to be dissected.

In contrast with labor-intensive and time-consuming experiments, computational prediction of lysine acetylation sites from protein sequences is also helpful to generate highly useful information for further experimental consideration. In 2006, we used 246 non-redundant lysine acetylation sites of 89 proteins as the training data set, and developed the first tool of PAIL for accurately predicting acetylation sites in proteins^16^. Later, Basu *et al*. prepared two training data sets containing 51 and 73 known acetylation sites respectively, and designed an alternative software package of PredMod^17^. In 2010, Gnad *et al*. compiled a much larger training data set with 3,600 human lysine acetylation sites from a large-scale study^7^, and adopted the support vector machines (SVMs) algorithm to predict acetylation sites^18^. To date, there have been at least a dozen of additional computational programs constructed for the accurate prediction of general lysine acetylation sites, such as LysAcet^19^, N-Ace^20^, EnsemblePail^21^, BPBPHKA^22^, PLMLA^23^,^24^, PSKAcePred^25^, KAcePred^26^, LAceP^27^, SSPKA^28^, AceK^29^, iPTM-mLys^30^ and KA-predictor^31^. However, none of them can predict HAT-specific sites. In 2012, Li *et al*. collected 267 and 82 sites modified by CBP/p300 and GCN5/PCAF HATs, respectively^11^,^32^. Using this training data set, they developed the first tool of ASEB to accurately predict HAT- or KAT-specific acetylation sites in the family level^11^,^32^. They further predicted and experimentally validated that MBD1 and MTA1 are exclusively acetylated by p300 but not PCAF, whereas DNA polymerase β (Pol-β) and DDB1 are specifically modified by PCAF but not p300^11^.

In this study, we aimed to develop a highly useful tool to predict HAT-specific lysine acetylation sites in the individual HAT level. First, we manually collected 702 experimentally identified HAT-specific sites of 205 proteins for seven well-characterized HATs, including CREBBP, EP300, HAT1, KAT2A, KAT2B, KAT5 and KAT8. In our data set, there were 544 and 158 HAT-specific acetylation sites in 160 human and 45 non-human proteins, respectively. A previously established algorithm of Group-Based Prediction System (GPS)^33^ was adopted and further improved for training a computational model for each HAT, by using human HAT-specific sites as the training data set. Then GPS-PAIL was constructed, whereas its prediction accuracy was critically evaluated with the leave-one-out (LOO) validation and *n*-fold cross-validations. We also compared GPS-PAIL with the existing tool of ASEB^11^,^32^, using non-human HAT-specific sites as a testing data set. We also used GPS-PAIL to perform a large-scale prediction of potential HATs for acetylation sites identified from high-throughput experiments in eukaryotes. Both online service and local packages of GPS-PAIL were implemented and could be accessed at http://pail.biocuckoo.org/.

## Results

### Sequence preferences around different types of HAT-specific acetylation sites

From the scientific literature and public data resources^11^,^15^,^32^, we totally collected 702 non-redundant HAT-specific acetylation sites of 205 protein protein substrates for seven HATs (Table 1, Supplementary Tables S1 and S2). The numbers of collected substrates and acetylation sites were summarized for each HAT, whereas the keywords used for searching HAT-specific acetylation sites were also present (Table 1). For convenience, the standard gene names in UniProt database^34^ were adopted. CREBBP and EP300, usually called as CBP and p300, belong to the p300/CBP family^1^,^10^. HAT1, KAT2A and KAT2B, also named as KAT1, GCN5 and PCAF, are key members of the GNAT family^1^,^3^,^12^. Also, KAT5 and KAT8, also called as Tip60 and MOF/MYST1, are essential HATs of the MYST family^3^,^35^.

**Figure 1:**
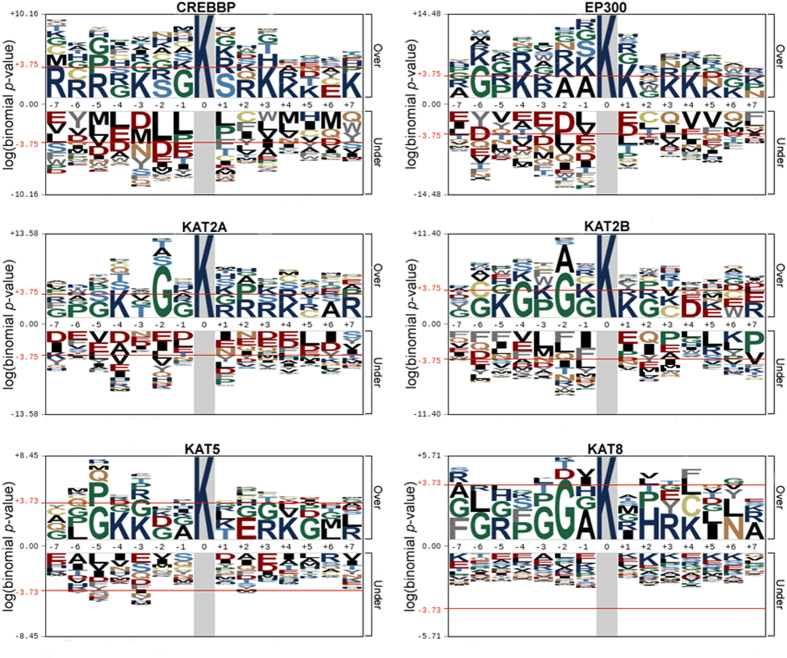
The amino acid frequencies of different types of HAT-specific lysine acetylation sites were analyzed and visualized by pLogo^36^.

Previously, it was demonstrated that different types of HATs can acetylate overlapping but distinct substrates^1^,^11^,^12^. For example, both CREBBP and KAT2B acetylate Ku70 at K542 *in vivo* to inhibit the Bax-mediated apoptosis^12^, whereas several proteins such as MBD1 and MTA1 are preferentially acetylated by p300 but not PCAF^11^. Thus, different HATs exhibit mutual but still distinct specificity for the substrate recognition, and we speculated whether there are potentially different sequence preferences around different types of HAT-specific sites. To address this problem, here we used pLogo^36^, a convenient tool for the visualization of sequence logos, to analyze the amino acid occurrence around different types of HAT-specific sites (Fig. 1). The sequence logo of HAT1 was not drawn due to the data limitation.

From the results, although the K residue is significantly over-represented in +3, +4 and +5 positions for both CREBBP- and EP300-specific acetylation sites, the G and S residues are enriched in −1 and +1 positions for CREBBP, whereas A and K residues prefer to occur at −1 and +1 positions for EP300, respectively (Fig. 1). For the GNAT family, a G residue preferentially occur at −2 position for both KAT2A and KAT2B, while the K and G residues are over-represented at −4 positions of KAT2A and KAT2B, respectively (Fig. 1). In addition, the residues of G, K and K prefer to occur at −5, −4 and −3 positions of KAT5-specific sites, while the residues of G, G and A preferentially occur at −3, −2 and −1 positions of KAT8-specific sites. However, the R and K residues are enriched at +3 and +4 positions for both KAT5 and KAT8, respectively (Fig. 1). Taken together, our results demonstrated that different types of HAT-specific sites have considerably similar but distinct sequence preferences.

### Development of GPS-PAIL for the prediction of HAT-specific lysine acetylation sites

Since different HATs have distinct sequence specificities for the substrate modifications, here we aimed to develop a highly useful tool to predict HAT-specific acetylation sites from protein sequences, and improved a previously established algorithm of GPS 2.2^33^ to train a computational model for each HAT, respectively. We used 544 human HAT-specific acetylation sites of 160 protein substrates as the training data set. For a convenient usage, both online service and stand-alone packages of GPS-PAIL were provided, with a user-friendly interface. GPS-PAIL can predict HAT-specific acetylation sites for seven HATs including CREBBP, EP300, HAT1, KAT2A, KAT2B, KAT5 and KAT8.

**Figure 2:**
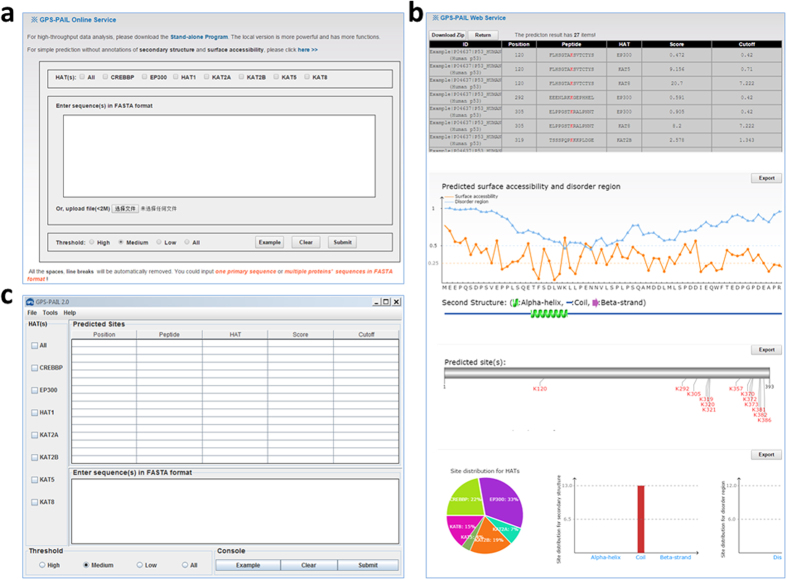
The user interface of GPS-PAIL online service and local packages. (**a**) As an example, the protein sequence of human p53 can be directly inputted for the prediction of HAT-specific acetylation sites. (**b**) The detailed predictions will be shown in a tabular format, while additional information such as surface accessibilities, disorder regions and secondary structures will be predicted and presented. A brief summarization of predicted sites will be also shown. (**c**) For predicting multiple protein sequences, the local packages can be downloaded and used with a higher speed.

The online service of GPS-PAIL was implemented in PHP and JavaScript. Also, two web services, IUPred^37^ and NetSurfP^38^ were integrated for the prediction of protein structural features, such as disorder regions, secondary structures and surface accessibilities. The website of GPS-PAIL was extensively tested on various web browsers such as Internet Explorer, Mozilla Firefox and Google Chrome to provide a robust service. For the usage of GPS-PAIL, here we chose the protein sequence of human p53 as an example (Fig. 2). The input of the online service contained three parts, including the HAT types, the protein sequences, and the thresholds (Fig. 2a). One or multiple HATs can be selected by clicking the checkboxes, while four threshold options including “High”, “Medium” and “Low” and “All” were provided in the lower panel. In GPS-PAIL, the “High”, “Medium” and “Low” thresholds were selected with *Sp* values of ~95%, ~90 and 85%, respectively. The “All” option will generate a predicted score for each lysine residues with no stringency. One or multiple protein sequences can be directly input or uploaded through a sequence file in FASTA format. Furthermore, users can transfer to the “comprehensive” mode by clicking the “here >>” link, to perform the predictions of secondary structures and surface accessibilities of given proteins (Fig. 2a).

After starting the prediction, the website will be redirected into a waiting page and then transferred to the result page (Fig. 2b). The results of p53 contained four sequential parts, including the list of 27 predicted HAT-specific acetylation sites with the HAT information, predicted surface accessibilities and disorder regions, predicted secondary structures, and a summarization of the results. All the results can be downloadable through clicking the “Download” button (Fig. 2b). To ensure the stability of the online service, the input of protein sequences was limited with <2MB, while the large-scale computation can be performed through installing the stand-alone software packages, which were implemented in JAVA and supported for three major operation systems including Windows, Linux and Mac OS (Fig. 2c).

### Performance evaluation and a comparison with ASEB

**Figure 3:**
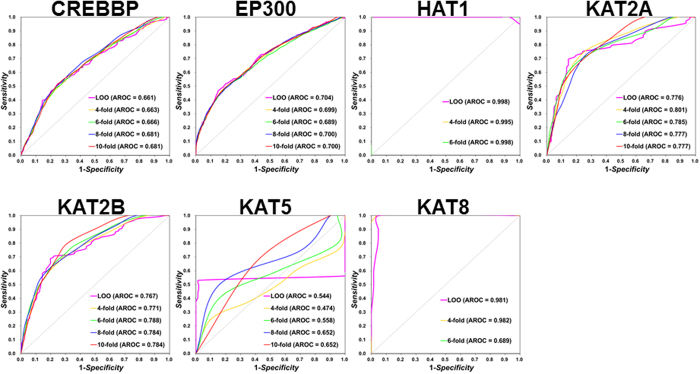
The LOO validation and *n*-fold cross-validations were performed for seven HATs including CREBBP, EP300, HAT1, KAT2A, KAT2B, KAT5 and KAT8. The known human HAT-specific sites were used for training. Due to the data limitation, only 4- and 6-fold cross-validations were carried out for HAT1 and KAT8. AROC values of the LOO validation and *n*-fold cross-validations were calculated.

To evaluate the prediction performance and robustness of GPS-PAIL, the training data set was used to perform the LOO validation and *n*-fold cross-validations. The receiver operating characteristic (ROC) curves were drawn, and the values of area under the curve (AUC) were calculated. Due to the data limitation, only 4- and 6-fold cross-validations were performed for HAT1 and KAT8, whereas 4-, 6-, 8- and 10-fold cross-validations were carried out for remaining HATs (Fig. 3). From the LOO results, AUC values are 0.661, 0.704, 0.998, 0.776, 0.767, 0.544 and 0.981 for CREBBP, EP300, HAT1, KAT2A, KAT2B, KAT5 and KAT8, respectively (Fig. 3). Thus, the prediction accuracies are generally satisfying except KAT5. In addition, the results of *n*-fold cross-validations are quite similar with the LOO results, suggesting the computational models were trained in a robust manner (Fig. 3).

Moreover, we used 158 non-human HAT-specific sites as an additional testing data set, and compared GPS-PAIL to ASEB, the first established tool for predicting HAT-specific sites in the family level^11^,^32^. For a justified comparison, we directly input the protein sequences of the testing data set to GPS-PAIL and ASEB to calculate the performances (Table 2). We fixed the specificity (*Sp*) values to be approximately identical and compared the sensitivity (*Sn*) scores. For convenience, the LOO results of GPS-PAIL on our training data set were also shown (Table 2). Although the accuracies of CREBBP and EP300 in GPS-PAIL were similar with the results of CREBBP/EP300 in ASEB, GPS-PAIL generated a much better performance against ASEB for KAT2A and KAT2B (Table 2). In addition, since more HATs were available for the prediction, GPS-PAIL is more applicable for further dissecting the signaling regulations of site-specific acetylation in proteins.

### Large-scale prediction of potential HATs for acetylomes in eukaryotes

**Figure 4:**
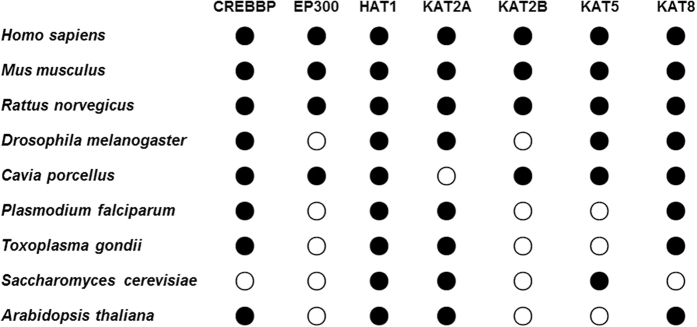
The potential orthologs of seven HATs among nine eukaryotic organisms were computationally identified with the approach of reciprocal best hits^39^. The existent HATs were marked with a black ball.

*Ab initio* prediction of HAT-specific acetylation sites directly from protein sequences will generate too many false positive hits. Thus, in this study we performed a systematic prediction of potential HATs for experimentally identified acetylation sites without the HAT information. Previously, we developed a comprehensive database of CPLM, containing 58,563 known lysine acetylation sites of 20,088 proteins from both eukaryotes and prokaryotes^15^. Because eukaryotic HATs were generally not conserved in prokaryotes, here we only predict potential HATs for eukaryotic acetylation sites. From CPLM, we totally obtained 44,850 sites in 15,898 proteins for nine eukaryotic species, including *Homo sapiens, Mus musculus, Rattus norvegicus, Drosophila melanogaster, Cavia porcellus, Plasmodium falciparum, Toxoplasma gondii, Saccharomyces cerevisiae*, and *Arabidopsis thaliana* (Supplementary Table S3). Before the prediction, we first determined the existence of potential orthologs of seven HATs across the nine organisms. We downloaded the proteome sequences of these species and pairwisely detected orthologs, using the strategy of reciprocal best hits (RBH)^39^. The orthologs of seven HATs were exactly identified and picked out if available (Fig. 4). From the results, we observed that the seven HATs were not equally conserved in eukaryotes. For example, all seven HATs are encoded in *Homo sapiens, Mus musculus* and *Rattus norvegicus*, whereas only HAT1, KAT2A/GCN5, and KAT5/ESA1 are conserved in *Saccharomyces cerevisiae* (Fig. 4). For each species, only detected HATs were selected for the large-scale predictions.

**Figure 5:**
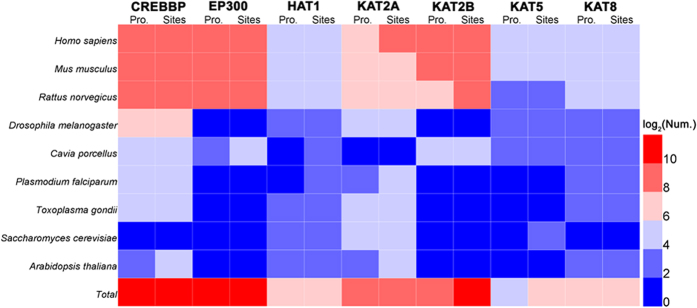
A summary of numbers of proteins and acetylation sites predicted with at least one HAT across nine eukaryotes. The heatmap was drawn with HemI^44^, and detailed statistics was shown in Supplementary Table S3.

To greatly reduce false positive predictions, the high threshold in GPS-PAIL was chosen. In the results, we predicted totally 4,344 acetylation sites of 2,764 protein substrates with at least one potential HAT, with an annotated coverage of 9.69% and 17.39% of all acetylation sites and proteins (Fig. 5 and Supplementary Table S3). For different species, the annotated coverage values ranged from 1.56% to 24.72% at the site level. For example, GPS-PAIL only predicted 67 sites of 46 substrates with at least one HAT from 4,284 un-annotated sites of 1,368 proteins in *Saccharomyces cerevisiae* (Fig. 5 and Supplementary Table S3). However, 14.20% and 24.72% of total acetylation sites were predicted with the HAT information in *Homo sapiens* and *Arabidopsis thaliana*, respectively (Fig. 5 and Supplementary Table S3). Thus, our results proposed that GPS-PAIL might be more efficient to predict HAT-specific acetylation sites in mammalians and plants.

**Figure 6:**
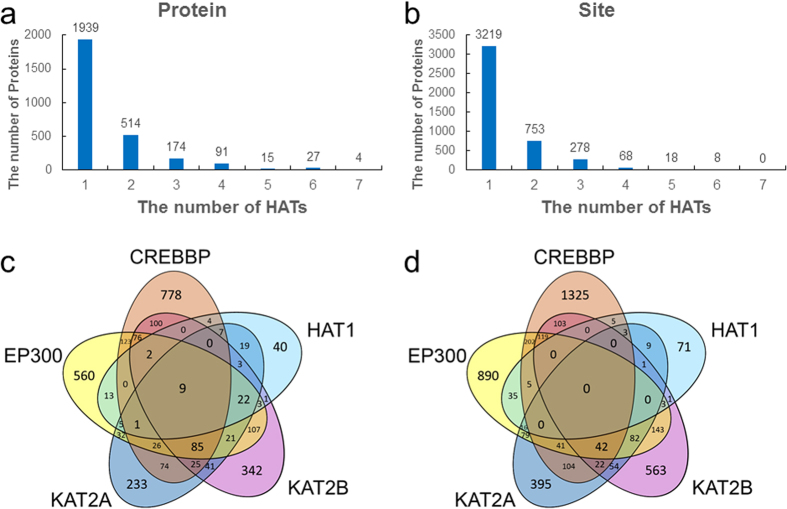
The distribution of predicted HAT-specific substrates and sites in nine eukaryotes. The number of potential HATs for (**a**) predicted proteins and (**b**) acetylation sites. The overlaps of (**c**) predicted substrates and (**d**) sites were shown for CREBBP, EP300, HAT1, KAT2A and KAT2B.

In addition, the distribution of numbers of protein substrates and sites modified by different types of HATs were analyzed (Fig. 6). Among 2,764 potential HAT-specific substrates, 1,939 proteins (70.15%) were predicted to be acetylated by only one HAT, whereas 514 substrates (18.60%) were predicted to be mutually modified by two HATs (Fig. 6a). Only 311 proteins (11.25%) might be regulated by over two HATs (Fig. 6a). In the site level, the results are similar that 3,219 (74.10%) and 752 (17.31) acetylation sites were modified by one and two HATs, respectively (Fig. 6b). The overlaps of predicted substrates and sites for CREBBP, EP300, HAT1, KAT2A and KAT2B were analyzed (Fig. 6c,d), while KAT5 and KAT8 were not included due to the data limitation from predictions. In the protein level, most of substrates were acetylated by only one HAT, while only nine proteins can be regulated by the five HATs (Fig. 6c). In the acetylation site level, the results were similar and no site can be modified by all the five HATs (Fig. 6d). Taken together, our large-scale analyses of predicted acetylated proteins and sites also demonstrated that different HATs recognize mutual but still distinct substrates. The detailed results of 2,764 proteins together with predicted sites and GPS-PAIL scores were shown in Supplementary Table S4.

## Discussion

HAT- or KAT-mediated acetylation at specific lysine residues of proteins is an essential PTM, conserved in both prokaryotes and eukaryotes, and plays a critical role in the regulation of numerous biological processes and cellular pathways^1^,^2^,^3^,^4^,^5^,^6^,^7^. Recent advances in the development of the state-of-the-art techniques in acetylomics have enabled to identify and quantify thousands of acetylation sites in a single run^5^,^7^,^14^. Although over 58,000 acetylation sites have been characterized in prokaryotic and eukaryotic species, the regulatory HATs of most of these sites still remain to be elucidated. Previously, we and others developed about 15 computational programs to predict general acetylation sites from protein sequences, with a satisfying accuracy^7^,^16^,^17^,^18^,^19^,^20^,^21^,^22^,^23^,^24^,^25^,^26^,^27^,^28^,^29^,^30^,^31^. However, the prediction of HAT-specific acetylation sites was still unavailable until the release of ASEB^11^,^32^, which clearly demonstrated that different types of HATs could modify distinct protein substrates^11^,^32^. Since ASEB only predicted HAT-specific sites in the family level, with only two predictors such as CBP/p300 and GCN5/PCAF, the prediction of specific acetylation sites for individual HATs is still a great challenge.

In this work, we first collected 702 known HAT-specific acetylation sites in 205 proteins for seven HATs including CREBBP, EP300, HAT1, KAT2A, KAT2B, KAT5 and KAT8, from the scientific literature and public data resources such as CPLM^15^ and ASEB^11^,^32^. The sequence preferences of different types of HAT-specific sites were analyzed, while the results demonstrated that different HATs recognize similar but considerably distinct sequence motifs for the substrate recognition. Using known human HAT-specific sites as the training data set, we further developed GPS-PAIL for the prediction of HAT-specific sites in the single HAT level, while both online service and local packages were implemented. We critically evaluated the prediction performance of GPS-PAIL by using the LOO validation and *n*-fold cross-validations. By a comparison with ASEB using non-human HAT-specific sites as an additional testing data set, GPS-PAIL exhibited at least a comparative accuracy. For HAT1 and KAT8, the values of *Pr, Sn* and *Sp* were all equal to 100% on the testing data set. However, the LOO results of HAT1 and KAT8 on the training data set didn’t reach an accuracy of 100% (Table 2). Because there were only 5 and 4 known HAT1- and KAT8-specific acetylation sites in the testing data set, we couldn’t conclude a perfect performance for the two HATs, and further evaluations still remain to be performed when more specific sites were experimentally identified.

Using GPS-PAIL, we performed a large-scale analysis to annotate potential HATs for known acetylation sites in nine eukaryotic organisms. Again, the large-scale prediction proposed that most of protein substrates and sites were acetylated by only one HAT, and the results further supported that different HATs recognize overlapping but still distinct substrates. We also carefully checked the literature and UniProt database^34^, and all known site-specific HAT-substrate relations (ssHSRs) in the prediction results were pinpointed (Supplementary Table S4). Previously, it was demonstrated that various functional features of proteins, such as gene ontology (GO) annotations and protein-protein interactions (PPIs), were beneficial for the prediction of kinase-specific phosphorylation sites^40^,^41^. In this work, the GO information was not used, because the functional diversity of HAT-specific acetylated substrates was high and no particularly significant GO terms were detected from the statistical enrichment analysis. However, the PPIs between HATs and substrates are potentially useful to reduce false positive predictions. From the STRING database^42^, the pre-integrated PPI data sets for nine species were retrieved, and the site-specific HAT-substrate relations with or without PPIs were shown (Supplementary Table S4). In the results, we observed there were 315 (5.23%) known ssHSRs with experimental evidences and 2,493 (41.41%) predicted ssHSRs with PPIs, respectively (Supplementary Table S4). Interestingly, we found 101 known ssHSRs without the PPI information (Supplementary Table S4). This is because interactions between HATs and substrates are usually transient and dynamic with a weak binding affinity, which might be difficult to be detected by standard PPI screenings or computational predictions.

For the future plan, we will continuously collect experimentally identified HAT-specific acetylation sites if available in the literature. Undoubtedly, a larger training data set will generate a more accurate performance for the prediction. Also, we will further refine and improve the prediction algorithm. For example, we recently developed GPS-SUMO for the prediction of sumoylation sites and SUMO-interaction motifs from protein sequences, with an enhanced version of GPS algorithm^43^. Currently, the GPS algorithm is still under improvement, and we will test the accuracy of the latest version of GPS algorithm on the prediction of HAT-specific acetylation sites. Taken together, in this study we developed an efficient tool GPS-PAIL to predict HAT-specific acetylation sites for seven HATs, with a satisfying accuracy. The prediction results of potential HATs for known acetylation sites in eukaryotes from the large-scale analysis can also serve as a useful data resource for further experimental consideration.

## Methods

### Data collection and preparation

First, we collected experimentally identified HAT-specific lysine acetylation sites from the scientific literature if available. For each known HAT^2^,^3^, we used its standard gene name, protein name or aliases together with the keyword of “acetylation” to search the PubMed database (Table 1). For example, multiple keyword combinations such as “’CREB-binding protein’ acetylation”, “CREBBP acetylation” and “CBP acetylation” were used to search CREBBP-specific acetylation sites, whereas “KAT5 acetylation”, “Tip60 acetylation” and “HTATIP acetylation” were used to find KAT5-specific sites (Table 1). The known HAT-specific sites in CPLM database^15^ and ASEB training data set^11^,^32^ were also integrated. Only HATs with at least five known specific sites were reserved for the further analysis, and their standard gene names from the UniProt database^34^ were adopted. Then we mapped all HAT-specific substrates to the primary protein sequences downloaded from the UniProt database^34^, and pinpointed the exact acetylation position. The redundancy was cleared, and the final data set contained 702 unique HAT-specific acetylation sites in 205 proteins, including 544 human acetylation sites in 160 proteins (Supplementary Table S1) and 158 non-human sites of 45 proteins (Supplementary Table S2).

In this study, the human HAT-specific sites were adopted for training, while non-human sites were used as an additional data set to test the prediction performance. For the preparation of the training data set, we defined an *acetylation site peptide* ASP(*m, n*) as an acetyllysine amino acid flanked by *m* residues upstream and *n* residues downstream. For each HAT, its experimentally identified acetylation sites were taken as positive data (+), whereas all the other non-acetylated lysine residues in the same proteins were regarded as negative data (−). The training and testing procedures were independently performed for each HAT. For the large-scale prediction of HAT-specific acetylation sites in eukaryotes, we totally obtained 44,850 known but un-annotated acetylation sites in 15,898 proteins of nine species from CPLM^15^ (Supplementary Table S3).

### Performance evaluation

As previously described^33^, three measurements of *Sn, Sp* and precision (*Pr*) were adopted to evaluate the prediction performance. The three measurements were defined as equation (1), (2), and (3):


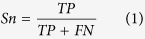



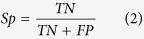



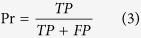


The LOO validation and 4-, 6-, 8- and 10-fold cross-validations were performed. The ROC curves were drawn and AROC values were calculated.

### Algorithm

Previously, we developed the GPS 2.2 algorithm for the prediction of APC/C recognition motifs such as D-boxes and KEN-boxes proteins^33^. The algorithm comprises two major parts, including the scoring strategy and performance improvement.

In the former part, based on the basic hypothesis of similar short peptides exhibiting similar biochemical properties with similar functions, we used an amino acid substitution matrix, e.g., BLOSUM62, to evaluate the similarity between two ASP(*m, n*) peptides of *A* and *B* as equation (4):


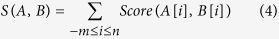


*Score*(*A*[*i*], *B*[*i*]) represents the substitution score of the two residues of *A*[*i*] and *B*[*i*] in the amino acid substitution matrix at the position *i*. If *S*(*A, B*) <0, we redefined it as *S*(*A, B*) = 0. For each HAT, a given ASP(*m, n*) was then pairwisely compared with each of its known specific acetylation sites to calculate the similarity score. The average value of the substitution scores was taken as the final score for the HAT.

The second part comprises three distinct steps, including motif length selection, weight training and matrix mutation. To monitor the performance improvement, here we fixed the *Sp* at 90% and compared *Sn* values of the LOO validation.

#### Motif length selection

In this step, the combinations of ASP(*m, n*) (*m* = 1, …, 30; *n* = 1, …, 30) were exhaustively tested, while the optimized combination of ASP(*m, n*) was determined based on the highest LOO result for each HAT, separately.

#### Weight training

Since different positions can provide different contributions to modification specificity, we refined the substitution score between the two ASP(*m, n*) peptides *A* and *B* was as equation (5):


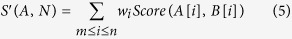


The *w*_*i*_ value denotes the weight of position *i*. Again, if *S′*(*A, B*) < 0, we redefined it as *S′*(*A, B*) = 0. Initially, the weight of each position in ASP(*m, n*) was take*n* as 1. Then we randomly picked out a weight of any position for +1 or −1, and adopted the manipulation if the LOO performance was increased. The process was continued until the *Sn* value was not increased any longer.

#### Matrix mutation

The aim of this step is to generate an optimal or near-optimal scoring matrix. BLOSUM62 was chosen as the initial matrix, and the LOO performance was calculated. Then we improved the *Sn* though randomly picking out an element of the BLOSUM62 matrix for +1 or −1. The process was repeated until convergence was reached.

During the training, the order of the three steps in performance improvement can be shuffled. To improve the training efficiency, we adopted the simulated annealing (SA) algorithm to optimize the parameters for the steps of Weight Training and Matrix Mutation.

### The PPI data sets

The PPIs together with corresponding protein sequences of nine species were downloaded from the STRING database (Version 10, http://string-db.org), which is an integrative data resource for both physical and functional associations among proteins in over 2,000 organisms^42^. Totally, we obtained 28,386,035 pairs of PPIs in nine species, including 4,274,001, 5,109,107, 5,319,621, 2,176,849, 2,340,229, 2,575,257, 332,297, 939,998 and 5,318,676 PPIs from *Homo sapiens, Mus musculus, Rattus norvegicus, Drosophila melanogaster, Cavia porcellus, Plasmodium falciparum, Toxoplasma gondii, Saccharomyces cerevisiae* and *Arabidopsis thaliana*, respectively.

## Additional Information

**How to cite this article**: Deng, W. *et al*. GPS-PAIL: prediction of lysine acetyltransferase-specific modification sites from protein sequences. *Sci. Rep.* **6**, 39787; doi: 10.1038/srep39787 (2016).

**Publisher's note:** Springer Nature remains neutral with regard to jurisdictional claims in published maps and institutional affiliations.

## Supplementary Material

Supplementary Tables

## Figures and Tables

**Table 1:** A summary of the numbers of acetylated substrates and sites for seven types of HATs curated from the literature.

HAT	Number		Keywords
	Substrate	Site	
CREBBP	71	248	CREB-binding protein acetylation; CREBBP acetylation; CBP acetylation
EP300	138	496	EP300 acetylation; P300 acetylation
HAT1	12	15	KAT1 acetylation; HAT1 acetylation
KAT2A	24	69	KAT2A acetylation; GCN5 acetylation; GCN5L2 acetylation
KAT2B	39	109	KAT2B acetylation; PCAF acetylation
KAT5	15	32	KAT5 acetylation; HTATIP acetylation; TIP60 acetylation
KAT8	8	10	KAT8 acetylation; MOF acetylation; MYST1 acetylation

The keywords used to search PubMed for the collection of HAT-specific acetylation sites were shown.

**Table 2:** Comparison of GPS-PAIL with ASEB^11^,^32^, by using 158 non-human HAT-specific sites of 45 proteins as the testing data set.

	HAT	Positive^*a*^	Negative	*Pr*	*Sn*	*Sp*
GPS-PAIL (LOO)	CREBBP	167	1719	16.12	38.32	80.63
	EP300	411	3525	22.81	50.61	80.03
	HAT1	10	110	62.50	100.00	94.55
	KAT2A	32	265	30.56	68.75	81.13
	KAT2B	69	954	17.17	49.28	82.81
	KAT5	28	531	39.29	39.29	96.80
	KAT8	6	177	33.33	83.33	94.35
GPS-PAIL (Testing)	CREBBP	81	405	28.32	39.51	80.00
	EP300	85	605	25.85	44.71	81.98
	HAT1	5	33	100.00	100.00	100.00
	KAT2A	37	430	19.23	54.05	80.47
	KAT2B	40	133	47.92	57.50	81.20
	KAT5	4	7	100.00	100.00	100.00
	KAT8	4	139	36.36	100.00	94.96
ASEB (Testing)	CREBBP/EP300	107	771	22.61	42.06	80.03
	KAT2A/KAT2B	73	522	23.08	41.10	80.84

For convenience, the LOO results of GPS-PAIL on our training data set were also provided. *a*. Positive, the number of positive sites.
